# How to Optimize Deimplementation of Sentinel Lymph Node Biopsy?

**DOI:** 10.1155/2024/7623194

**Published:** 2024-05-24

**Authors:** Ida Dragvoll, Anna M. Bofin, Håvard Søiland, Monica Jernberg Engstrøm

**Affiliations:** ^1^Department of Clinical and Molecular Medicine, Faculty of Medicine and Health Sciences, Norwegian University of Science and Technology (NTNU), Trondheim 7491, Norway; ^2^Department of Breast and Endocrine Surgery, St. Olav's Hospital, Trondheim University Hospital, Pb 3250 Torgarden, Trondheim 7006, Norway; ^3^Department of Research, Stavanger University Hospital, Pb 8100, Stavanger 4068, Norway; ^4^Department of Clinical Science, University of Bergen, Jonas Lies vei 87, Bergen 5021, Norway

## Abstract

**Background:**

The omission of sentinel lymph node biopsy in low-risk elderly breast cancer patients has been introduced in several guidelines. Despite evidence to support its safety, this recommendation has not been implemented by many clinicians. We have examined two aspects of this recommendation that may explain why sentinel lymph node biopsy continues to be performed in most of these patients. Firstly, we quantified the proportion of patients diagnosed with axillary metastases postoperatively. Secondly, we examined adherence to antihormonal therapy in the same group of patients.

**Methods:**

In this single-centre retrospective cohort study, the study population comprised 98 patients with breast cancer. Patients were aged ≥70 years and diagnosed with hormone receptor positive breast cancers less than 20 mm (T1). All patients underwent surgery and were subsequently prescribed five years of adjuvant antihormonal treatment.

**Results:**

Axillary lymph node metastases, as confirmed by the postoperative histology report, were seen in 36.3%. Nonadherence was seen in 33.7% of the patients. Primary nonadherence, that is, patients that never collect their first or subsequent prescriptions at the pharmacy, comprised 11.2% of the total study population.

**Conclusion:**

The high proportion of axillary metastases demonstrated suggests that clinical examination of the axilla alone is not sufficient in the preoperative assessment of the axilla. The less-than-optimal adherence rates show that adherence in these patients cannot be taken for granted. We suggest that these factors reflect some of the reluctance among clinicians to omit the sentinel lymph node procedure in these patients.

## 1. Introduction

Focusing on the challenge of overtreatment in breast cancer has led to the deescalation of previously well-established therapies. Overtreatment can be defined as treatment that does not convey a benefit to the patient but rather may cause harm. Moreover, unnecessary treatment leads to unnecessary costs for health care systems [1–3]. This pervasive problem has gained increasing attention in recent years. Minimising overtreatment is of particular importance in elderly breast cancer patients where comorbidities and frailty frequently occur [4]. The population is aging [5], and over 30% of new breast cancer diagnoses in the US occur in women ≥70 years [6].

The *Choosing Wisely Campaign* was established in the US in 2012 with a goal to reduce unnecessary medical testing and treatment by engaging clinicians and patients in conversations about the topic. Since its inception, many countries around the world have adapted and implemented the campaign [7, 8]. The *Choosing Wisely* initiative published the following recommendation in 2016: “Don't routinely use sentinel node biopsy in clinically node negative women ≥70 years of age with early stage hormone receptor positive, HER-2 negative invasive breast cancer.” This has later been reaffirmed in updated versions in 2019, 2020, and 2021 [9]. The same recommendation was made by the American Society of Clinical Oncology (ASCO) in 2021 [10]. Both recommendations state that these patients should be treated with adjuvant antihormonal treatment. These recommendations were made mainly based on the findings in the Cancer and Leukaemia Group B 9343 (CALBG 9343) clinical trial published in 2004 [11] with a follow-up study in 2013 [12]. These trials demonstrated similar survival in women who received radiotherapy and tamoxifen compared to those who received tamoxifen only following breast conserving surgery. Omitting SLNB, and therefore radiotherapy, has consequently been deemed safe by the *Choosing Wisely Campaign* and ASCO. Other studies have showed concordant results [13–16].

Although there is extensive evidence showing that omitting sentinel lymph node biopsy (SLNB) and radiotherapy in these patients is safe, the implementation of this recommendation remains to be embraced by most clinicians treating breast cancer [13, 17–20]. Retrospective data have shown that between 68% [21] and over 80% [22, 23] of patients eligible for omission of SLNB according to the abovementioned recommendations still undergo the procedure. The explanation for this is multifactorial showing that effective deescalation relies on more than evidence from clinical trials [24–26].

We propose that there are two prerequisites to this recommendation that may explain its unsuccessful implementation. Firstly, preoperative examination of the axilla must verify the absence of axillary metastases (cN0), and secondly, the patient is expected to adhere to adjuvant antihormonal treatment for five years after diagnosis. The main aims of the present study were to quantify the proportion of patients in whom axillary lymph node metastases are detected postoperatively and to examine adherence to antihormonal treatment in this specific group of patients. Furthermore, we examine how adherence and axillary lymph node status relate to survival for these patients.

## 2. Materials and Methods

The study population comprised all patients aged ≥70 years at the time of diagnosis who underwent surgery for estrogen receptor positive breast cancer at St. Olav's University Hospital in the period 01.01.2004 to 31.12.2013. Information regarding age, tumour characteristics, lymph node status, and treatment modalities were collected from the hospital medical records. Patients underwent breast conserving treatment (BCT) or mastectomy of the tumour with SLNB and/or axillary lymph node dissection (ALND). Tumours were confirmed to be estrogen receptor positive and less than 20 mm in diameter (T1 tumours) according to the postoperative pathology report. All patients were prescribed adjuvant antioestrogen treatment for five years. Patients with tumours that were of histological grade 1 and TI were excluded from the material if national guidelines operative at the time of diagnosis did not recommend antihormonal treatment. Also, patients with estrogen receptor low-positive tumours (≥1% < 10%) diagnosed prior to 2011 were excluded as these patients were not recommended antihormonal treatment at that time. Patients who received neoadjuvant treatment were excluded. All patients were followed for at least five years.

Preoperative evaluation of the axilla consisted of clinical examination and axillary ultrasound. All SLNB were performed using dual tracer with radioactive isotope and blue dye. Lymph node positivity was defined as any detected metastases within a lymph node. During the study period, frozen sections were performed on all lymph nodes removed during the SLNB procedure. An axillary dissection was performed if any metastases ≥2 mm were detected.

Detailed information regarding the prescribed antihormonal treatment was retrieved from the Norwegian Prescription Database. Adherence was determined by the medical possession ratio (MPR). MPR was measured as a fraction where the numerator was the total amount of tablets dispensed. The denominator was five years. Patients were defined as nonadherent if the MPR was <80%. For those who died during the five-year course of antihormonal treatment, the denominator was adjusted to the length of time they were alive after commencing the treatment [27].

Nonadherent patients were further subclassified into primary nonadherent (PNA) or secondary nonadherent (SNA). Primary nonadherence occurs when a patient is prescribed a treatment but fails to collect the first and subsequent prescriptions at the pharmacy. Secondary nonadherence is defined as failure to take the medication as prescribed after the first prescription has been collected, with a measured MPR of <80% [28–30].

Overall survival and BCSS were assessed using Kaplan–Meier curves. Statistical analyses were performed using SPSS version 28.

## 3. Results

**Figure 1:**
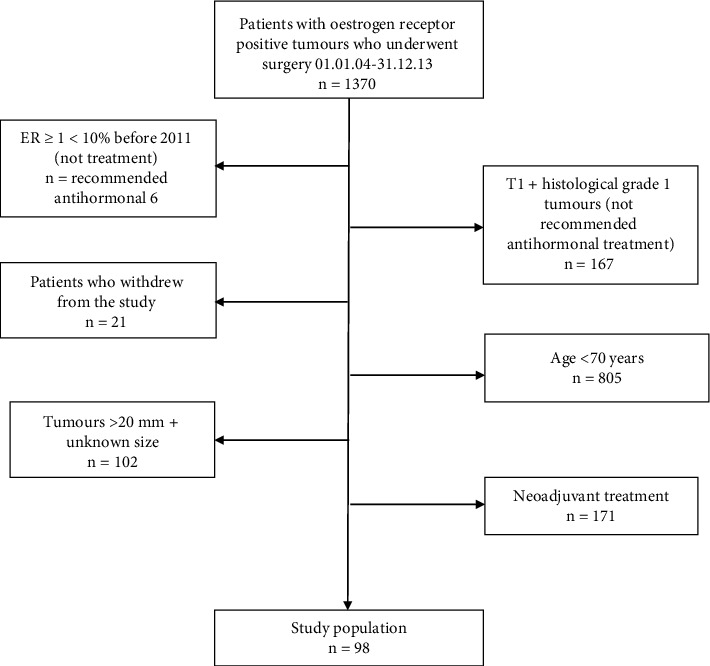
Flow chart of study population.

A total of 98 patients (Figure 1), 97 females and one male, were included in the study. The median age at diagnosis was 77 (range 70–93). Of the total study population, 66.3% (65/98) were adherent, 11.2% (11/98) were PNA, and 22.4% (22/98) were SNA to the prescribed antihormonal treatment (Table 1). No axillary lymph node metastases (pN0) were found in 59.2% (58/98), 1–3 axillary metastases were found in 25.5% (25/98), and ≥4 in 8.2% (8/98) of the study population.

At the end of the follow-up, 12.2% (12/98) of patients had died due to breast cancer, 30.6% (30/98) had died of causes other than breast cancer, and 57.1% (56/98) were alive (Table 2).

**Figure 2:**
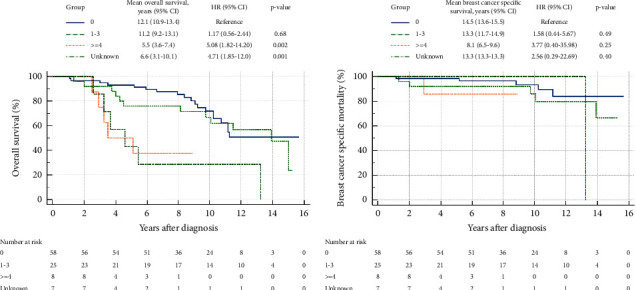
Overall survival (a) and breast cancer specific survival (b) according to the number of axillary lymph node metastases.

**Figure 3:**
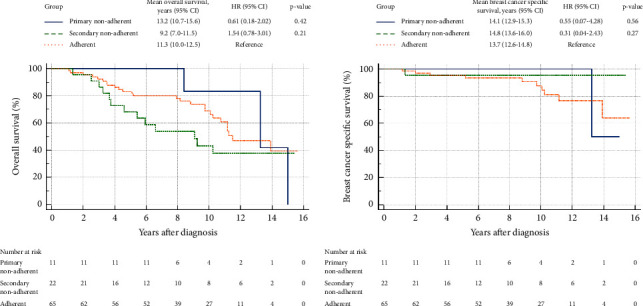
Overall survival (a) and breast cancer specific survival (b) according to adherence status.

Figure 2 shows that the PNA patients tend to have better overall survival (OS) than the adherent patients (HR 0.61, 95% CI 0.18–2.02). SNA patients may be more likely to have worse OS compared to the adherent patients (HR 1.5, 95% CI 0.78–3.01).  Figure 3 shows a worsening survival with an increasing number of axillary metastases. Overall survival was poorer for those with ≥4 metastases (HR 5.1, 95% CI 1.8–14.2) compared to patients with no axillary metastases.

## 4. Discussion

In this study, we found that about one-third of the patients had axillary lymph node metastases as documented on the postoperative pathology report. Nonadherence to the prescribed treatment was seen in approximately one-third of patients, and of these, one-third failed to collect their first and subsequent prescriptions.

In the CALBG 9343-trial [11], the majority of women had estrogen receptor positive T1 tumours, and all were ≥70 years of age. A total of 636 women were randomized to either breast conserving treatment with adjuvant radiotherapy and tamoxifen or breast conserving treatment and tamoxifen alone. In both arms, about one-third of the patients underwent axillary lymph node dissection (ALND). The study concluded that the two arms had similar survival and a similar risk of distant metastases. The only significant difference between the two groups was an increased risk of local or regional recurrences at five years for those who did not receive radiotherapy (4% risk for those not receiving radiotherapy and 1% risk for those receiving tamoxifen and radiotherapy). A long-term follow-up study of the CALBG 9343-trial published in 2013 [12] showed similar results. This study also demonstrated that the slightly increased risk of local recurrences in those treated without radiotherapy did not result in decreased survival.

Of the 91 patients in our study with known axillary status, 36.3% (33/91) had axillary metastases confirmed on the postoperative pathology report. According to the abovementioned guidelines, omitting SLNB is only done if the preoperative clinical examination of the axilla reveals no signs of metastases. It has been shown that the clinical evaluation of the axilla is inaccurate with regard to detecting axillary lymph node metastases [31, 32]. Similar to our results, a study of 5125 patients with negative preoperative clinical examinations of the axilla who underwent axillary dissection revealed that 34% had axillary metastatic disease according to the histopathological report [33]. A further study among women with no palpable axillary lymph nodes revealed that 32% had axillary metastases on histopathological examination [34].

Preoperative clinical data were unavailable to us. However, considering the rather low detection rates of axillary metastases by clinical examination alone, it is highly likely that metastases would have been missed. With the concomitant use of axillary ultrasound, a higher number of metastases would be detected. The use of both clinical examination and axillary ultrasound should therefore be encouraged [35, 36]. Some argue that axillary ultrasound should replace clinical examination of the axilla as they show that ultrasound has significantly higher sensitivity and accuracy than clinical examination alone [37]. However, ultrasound of the axilla is highly examinator dependent with a positive predictive value ranging from 77 to 83% and a negative predictive value between 52 and 67% [38, 39]. Furthermore, the necessity of axillary ultrasound in this setting can be questioned. The ACOSOG Z0011 study has changed the management of axillary metastases allowing for the omission of ALND in patients with one to two positive sentinel lymph nodes. Inclusion criteria in this study allowed patients with no palpable axillary lymphadenopathy eligible without the use of axillary ultrasound [40]. The SOUND trial randomized patients with T1 tumours and a negative axillary ultrasound to undergo SLNB or not. Of those who underwent SLNB, 13.7% had axillary metastases. However, there was no difference in distant disease-free survival or adjuvant treatment recommendations between the two groups [16]. Nevertheless, it must be acknowledged that in current clinical practice, axillary ultrasound is largely regarded to be standard-of-care in the preoperative evaluation [41].

As expected, we show that better survival is seen in patients with the fewest axillary lymph node metastases. Furthermore, studies have shown that a positive SLNB does affect adjuvant treatment decisions [42–44]. Radiation therapy in the CALBG 9343-trial was delivered as whole breast irradiation including axillary level I and II over 25 daily fractions. Due to the relatively low numbers of axillary metastases in these patients and the findings of similar survival for those who did, or did not, undergo axillary evaluation in the CALBG 9343-trials, the guidelines have deemed it safe to omit SLNB. Omitting SLNB means missing the opportunity to treat potential axillary metastases. However, the PRIME II study concluded that it is safe to omit radiotherapy in selected low-risk patients undergoing breast conserving surgery [14]. Omitting radiotherapy altogether might be difficult to accept for many clinicians, especially if the patient is in her early 70s with no or few comorbidities. Some argue that a more appropriate way to deescalate would be to give partial breast radiation to those with unknown axillary status [45]. However, others would be reluctant if axillary status is unknown for fear of missing undiagnosed axillary metastases [46].

Despite evidence that survival is not affected by the omission of SNLB, it might still be difficult to accept forgoing a well-established procedure. SLNB is considered to be safe and accurate and is associated with few complications [47, 48]. It is a relatively minor procedure performed at the same time as the primary breast surgery.

The present study shows that 33.7% (33/98) of the study population were nonadherent at five years of follow-up. Patients in the present study were subclassified into PNA or SNA. Although the numbers are low, over 10% of the patients were PNA. That is, they never initiated the antihormonal treatment they were prescribed. We suspect that these patients' lack of appreciation of the need for antihormonal treatment may be largely due to poor patient-doctor communication. If deescalating in surgery occurs by omitting the SLNB in elderly breast cancer patients, it is highly important that time is spent with the patient explaining the rationale for prescribing the antihormonal treatment in order to minimize both PNA and SNA. We observed that more than 20% of the patients were SNA. That is, they initiated the treatment, but for some reason, they failed to reach a MPR of ≥80%. The main reason for becoming nonadherent to antihormonal treatment is its side effects [49–51]. As many patients experience troublesome side effects affecting their quality of life, some of these patients choose to discontinue the treatment [52, 53]. Treating these side effects will help patients remain adherent to the treatment [52, 54]. We therefore suggest that in the case of deescalation by omitting SLNB, patients should be followed closely in order to uncover troublesome side effects or other reasons for nonadherence.

Various factors have been shown to affect adherence to antihormonal treatment. Advanced age and low-risk disease are known predictors of poor adherence to antihormonal treatment [49, 55, 56]. This puts the patients included in this recommendation at a higher risk of nonadherence compared to many other breast cancer patients. Omitting SLNB could potentially be a motivating factor for remaining adherent to the antihormonal treatment. This would perhaps give higher adherence rates than in the current study. Adherence behaviour is a complex matter, and causes of nonadherence are often multifactorial [57, 58]. It is therefore important to address the issue of adherence early, that is, even before the treatment is commenced.

A prerequisite of the abovementioned guidelines is five years of adherence to antihormonal treatment. However, previous literature has documented that adherence to adjuvant antihormonal treatment is poor [49, 52, 59–62]. Nonadherence rates have been described in the range of 10.8% [63] to 55% [54]. This wide range is probably due to the lack of a uniform definition of adherence and also varying ways of measuring it [64–66]. Based on these figures, it is likely that some of the patients included in the CALBG 9343-trial also were nonadherent. Despite this, survival rates were good. One could therefore argue that adherence to antihormonal therapy is not of importance in this population. However, it is our opinion that as we strive to improve patient care, adherence will continue to be of importance and even better survival rates could have been achieved with optimal adherence. It is therefore of importance to carefully select patients who will benefit from the treatment and avoid unnecessary side effects in those who will have a minimal effect of the treatment. Furthermore, the population is aging [5], and as we see a shift towards increased use of oral anticancer agents taken at home, the impact of poor adherence is likely to become even more important in the years to come [67, 68].

We have previously shown that PNA patients have better prognosis compared to both adherent and SNA patients [61]. Similarly, in the subgroup of patients in the present study, PNA patients tend to have better survival than both the adherent and SNA patients. This adds a contribution to the discussion regarding the possible overtreatment of low-risk patients as the PNA patients had the best survival.

Over the past decades, deescalation of treatments in breast cancer patients has gained increasing attention. As more treatment options become available and our knowledge expands, the need to tailor treatments to each individual patient has become an integral part of modern breast cancer management. Deescalation will spare the patient for potential morbidity associated with treatment and simultaneously reduce expenditure for health care systems [3, 24, 69]. However, deescalation should be carried out with caution and should be based on solid clinical research. Furthermore, implementation of deescalation guidelines should be monitored closely, especially when these guidelines do not seem to be widely accepted by clinicians.

From a financial point of view, health services would benefit from the omission of SLNB [70]. In an already pressurized health care system, the omission of SNLB would free up resources, shorten waiting lists, and allow for resources to be directed elsewhere [46].

In modern breast cancer management, treatment recommendations are increasingly based on tumour biology rather than nodal status [69]. Guidelines based on chronological age might not be the optimal way of stratifying patients. In the future, stratifying patients according to the biomarker profile and comorbidity are likely to become the preferred option in order to determine who is eligible for the omission of SLNB [71, 72].

One of the strengths of this study is the quantification of adherence rates in this specific group of patients and the subdivision of nonadherent patients into PNA and SNA, thus further examining the adherence behaviour of these patients. However, there are several limitations to this study. It is a small and retrospective study. Data regarding whether a patient underwent SLNB and axillary dissection or axillary dissection only were unfortunately not available to us. Patients with HER-2 positive tumours or with unknown HER-2 status were included in this study. These patients could have benefitted from SLNB as proven metastases would have affected adjuvant treatment decisions. Furthermore, patients with pT1 and histological grade 1 tumours were not recommended antihormonal treatment according to the national guidelines operative in the study period. These patients were therefore excluded from the material leaving the study population somewhat skewed towards larger tumours and higher histological grades. Furthermore, molecular subtyping was not available in this study population.

Some of the abovementioned patients with pT1 and histological grade 1 tumours did receive antihormonal treatment. These patients were therefore included in the study. We find this interesting as this group of patients was not treated according to the guidelines operative at the time of diagnosis. This confirms that the guidelines often are mere recommendations and that clinicians in selected cases choose to treat patients on an individual basis and according to the preferences of the patients. This shared decision-making will provide optimal care for each patient [73]. We believe that the individual assessment of each patient, especially with regard to comorbidity, is one of the main reasons for deviation from the guidelines.

## 5. Conclusion

Despite the small size of this study, it underpins much of the reluctance among clinicians to omit SLNB in elderly low-risk breast cancer patients. While reluctance is both understandable and necessary, the presence of axillary metastases in these low-risk breast cancer patients has not been shown to affect survival. In the future, we suggest that the selection of patients eligible for the omission of SLNB should be personalised based on the principles of shared decision-making including biomarker profiling and assessment of comorbidities.

## Figures and Tables

**Table 1:** Patient characteristics.

	Total	Adherent	PNA	SNA
*N* (%)	98	65 (66.3)	11 (11.2)	22 (22.4)
Age at diagnosis				
70–79	66 (67.3)	52 (80)	6 (54.5)	8 (36.4)
≥80	32 (32.7)	13 (20)	5 (45.5)	14 (63.6)
T-stage				
pT1a	1 (1)	1 (1.5)	0	0
pT1b	15 (15.3)	8 (12.3)	6 (54.5)	1 (4.5)
pT1c	82 (83.7)	56 (86.2)	5 (45.5)	21 (95.5)
Histopathological type				
Ductal	74 (75.5)	50 (76.9)	8 (72.7)	16 (72.7)
Lobular	13 (13.3)	8 (12.3)	2 (18.2)	3 (13.6)
Other	10 (10.2)	7 (10.8)	1 (9.1)	2 (9.1)
Unknown	1 (1)	0	0	1 (4.5)
Histopathological grade				
Grade 1	14 (14.3)	9 (13.8)	1 (9.1)	4 (18.2)
Grade 2	65 (66.3)	42 (64.6)	9 (81.8)	14 (63.6)
Grade 3	17 (17.3)	13 (20)	1 (9.1)	3 (13.6)
Unknown	2 (2)	1 (1.5)	0	1 (4.5)
HER-2 status				
Negative	66 (67.3)	44 (67.7)	8 (72.7)	14 (63.6)
Positive	12 (12.2)	10 (15.4)	1 (9.1)	1 (4.5)
Unknown	20 (20.4)	11 (16.9)	2 (18.2)	7 (31.8)
Axillary lymph node metastases				
0	58 (59.2)	37 (56.9)	9 (81.8)	12 (54.5)
1–3	25 (25.5)	19 (29.2)	1 (9.1)	5 (22.7)
≥4	8 (8.2)	7 (10.8)	0	1 (4.5)
Unknown	7 (7.1)	2 (3.1)	1 (9.1)	4 (18.2)
Radiotherapy				
No	56 (57.1)	36 (55.4)	7 (63.6)	13 (59.1)
Yes	42 (42.9)	29 (44.6)	4 (36.4)	9 (40.9)
Chemotherapy				
No	94 (95.9)	61 (93.8)	11 (100)	22 (100)
Yes	4 (4.1)	4 (6.2)	0	0

**Table 2:** Axillary metastases and adherence according to patient status at the end of follow-up.

	Dead due to breast cancer	Dead due to other cause	Alive
*N* (%)	12 (12.2)	30 (30.6)	56 (57.1)
Axillary metastases			
0	5 (41.7)	14 (46.7)	39 (69.6)
1–3	5 (41.7)	7 (23.3)	13 (23.2)
≥4	1 (8.3)	4 (13.3)	3 (5.4)
Unknown	1 (8.3)	5 (16.7)	1 (1.8)
Adherence status			
PNA	1 (8.3)	2 (6.7)	8 (14.3)
SNA	1 (8.3)	12 (40)	9 (16.1)
Adherent	10 (83.3)	16 (53.3)	39 (69.6)

## Data Availability

Data that support the findings of this study are available from the Norwegian Prescription Database. Restrictions apply to the availability of these data, which were used under license for the current study and so are not publicly available. Patient data collected from the medical records in the hospital are not available due to patient confidentiality.

## Supplementary Materials

Supplementary Materials STROBE checklist. STROBE statement: checklist of items that should be included in reports of cohort studies.
